# 

**Published:** 1875-01

**Authors:** 


### TABLE OF CONTENTS.

### ORIGINAL COMMUNICATIONS.

A THEORY OF THE PATHOLOGY OF MALARIAL HÆMATURIA. *By B. M. Cromwell, M. D., Albany, Ga.* ........ 577

THE BRAIN (CEREBRUM) AS AN AFFERANT OR INCIDENT EXCITOR TO NERVOUS PERIPHERY, AND AS RELATED TO VOLITIONAL AND EMOTIONAL MOVEMENTS, ETC. *By E. F. Starr, M. D., Nacoochee, Ga.* ........ 583

MALARIAL HÆMATURIA IN HOUSTON COUNTY, GA. *By L. B. Alexander, M. D., Forsyth, Ga.* ........ 591

INTUSSUSCEPTION—DISTENSILE ENEMATA. *By B. C. Smith, M. D., Cold Water, Ga.* ........ 598

### CLINICS.

Eye and Ear Clinic Atlanta Medical College ........ 600

### ATLANTA ACADEMY OF MEDICINE.

Suspected Extra-Uterine Pregnancy—Phlegmasia Dolens, New Treatment—Discussion of its Pathology—New Instrument for Intra-Uterine Medication—Dysmenorrhœa with Catalepsy—Case of Dislocation of Spleen—New method in Hare-Lip—Peculiar Persistent Pain in the Feet—Urethral Stricture. 606

### OUR EXCHANGES.

The Physician and the God of Love (Poem) ........ 616

Esmarch's Method ........ 617

Erichsen on American Surgery ........ 618

The New Year, *Dr. N. S. Davis* ........ 619

Darwinism vs. Medicine ........ 620

Transfusion of Lamb's Blood—Results ........ 621

Hypodermic Ergot in Post-Partum Hemorrhage ........ 622

Improved Test for Sugar in Urine ........ 923

Pathology of Jaundice ........

Limit to Child Bearing ........

Should a Man with Gleet Marry? ........ 624

Mind Reading ........ 625

Some Things about Rain ........ 627

Subnitrate Bismuth—Adulteration ........

Foreign Bodies in the Err ........ 631

Don't Crowd (Poetical) ........

Displacement of Spleen ........

Boot Jelly and Shirt Coffee ........ 632

Hidden Penis ........

A Brave Surgeon ........ 633

Cold Powder ........

Whooping Cough ........ 634

### BIBLIOGRAPHICAL.

Von Ziemssen's Cyclopedia ........ 635

Toner's Sketch of Early American Medicine ........ 639

### EDITORIAL.

The Laborer Worthy of His Hire ........ 636

Registration of Births, Deaths and Marriages ........ 637

